# Self-Active Relaxation Therapy (SART) and Self-Regulation: A Comprehensive Review and Comparison of the Japanese Body Movement Approach

**DOI:** 10.3389/fnhum.2018.00021

**Published:** 2018-02-08

**Authors:** Russell S. Kabir, Yutaka Haramaki, Hyeyoung Ki, Hiroyuki Ohno

**Affiliations:** ^1^Graduate School of Education, Hiroshima University, Higashihiroshima, Japan; ^2^Department of Clinical Psychology, Hiroshima University, Higashihiroshima, Japan; ^3^Department of Psychology, Fukuoka Jo Gakuin University, Fukuoka, Japan; ^4^Department of Clinical Psychology, Fukuoka Jo Gakuin University, Fukuoka, Japan

**Keywords:** relaxation, self-regulation, disaster mental health, stress management, self-active relaxation therapy

## Abstract

Relaxation programs are known for their versatility, cost-effectiveness, and ability to help people obtain skills to regulate their mental states and promote and maintain health. Self-Active Relaxation Therapy (SART) is a body-oriented approach to psychological rehabilitation that grew out of the suite of movement tasks developed in the Japanese psychotherapy known as *Dohsa-hou*, or the body movement method. The program for SART is designed to stretch, twist, and release areas of the upper, lower, and whole body through a set of movements which are guided by the practitioner and performed “self-actively” by the client to empower them to learn to recognize points of tension in the body and act on their own to achieve a relaxed state. Numerous studies have showed that SART is associated with reduced negative mood states and enhanced body awareness. A short version of SART has been investigated as a psychological support salon activity for the elderly, mothers raising children, special needs students, and children adapting to school. The full program has also been applied in clinical settings to address or supplement treatments for psychological and developmental conditions, and longitudinally employed in community contexts to assist residents facing long-term disaster recovery circumstances in Japan. This paper reviews the research and applications of SART as a bodymind approach by critically examining evidence and research gaps for future studies, comparing it with techniques established in the literature, and positing a self-regulatory framework for SART as a tool to become aware of bodily states, regulate mood, and manage stress through the deliberate practice of relaxation.

## Introduction

Contemplative and bodymind practices are increasingly leveraged as salutary tools for mental and physical health management across the globe. Recent veins of research have challenged the widely propagated Western intuition of mind-body separation, often attributed to the work of Descartes, as invoking a distinction without a difference. Contemporary paradigms of the embodied position stipulated by Damasio (1994) and discerned by Craig (2002) show that psychological phenomena such as emotions and other internal states are brought to consciousness through a multisensory coordination of signals linked to homeostatic processing, also known as *interoception* (Tsakiris and Critchley, 2016). Prolonged exposure to the stress response can lead to allostatic consequences, and it is thought that stress may alter interoceptive signal processing as a result (Schulz and Vögele, 2015). The tools afforded to those employing contemplative and bodymind practices work to buffer against states of the body that are induced by prolonged exposure to the stress response, often by facilitating a sense of positive body awareness or interoceptive sensibility (Mehling et al., 2012; Mehling, 2016). In this manner, bodymind approaches can be considered ways to develop adaptive skills for self-regulation and health maintenance.

An original psychotherapy from Japan known as *Dohsa-hou*, or the “body movement method,” emerged from research in rehabilitation and extends the repertoire of these approaches as it focuses on universal bodily responses as nonverbal indicators of internal states (Dadkhah, 1998; Kubota, 2000; Fujino, 2012; Konno, 2016). Dohsa-hou was developed in 1966 by Gosaku Naruse from his work with youth dealing with cerebral palsy. Naruse (1973) observed that patients with cerebral palsy characteristically exhibit excess muscle tension in their body movements that incurs the loss of an intact sense of body ownership and exacerbates a cycle of frustration (Konno, 2016). This is purportedly due to a perceptually asynchronous relationship between their intended and resultant body movements, or a disconnect in motor prediction and motor resonance (Harada, 2016). In a series of eye-opening experiments, Naruse found that patients with cerebral palsy were able to move more fluidly in accordance with directed movement tasks under an induced state of hypnosis, revealing that elements of cognitive processing are involved in motor control (Naruse, 1973). Naruse formulated these observations into a theory known as Dohsa-hou, in which a coherence between the psychological and physiological process of movement is achieved when the client intends to move a body part, strives toward that goal, and realizes the movement they intended (Naruse, 1988, 1997). This discovery of a psychological component to the experience of body rigidity from cerebral palsy was a cornerstone for studies in the field and spurred new applications of “motor action training” tasks to various developmental and psychological conditions, to include schizophrenia, depression, anxiety, and others (Naruse, 1997; Kubota, 2000; Imura et al., 2016; Konno, 2016). It has been used to bolster self-control processes for athletes in Iran (Dadkhah, 1998), taught in workshops to facilitate post-traumatic coping for adults in Cambodia (Imura, 2016), pilot tested at a daycare center for children in Bulgaria (Chervenkova, 2015), and demonstrated in accredited camps held in South Korea, India, Malaysia, and Thailand (Harada, 2016; Harada and Teruta, 2016). These training camps have produced dramatic results in those with movement disorders, as they temporarily become able to perform intended movement tasks such as kneeling or standing after weeklong cooperative efforts with their certified trainer (Harada, 2016; for review: Chervenkova, 2017).

Dohsa-hou, while cogently described, continues to be a technique chiefly utilized in the context of rehabilitation psychology as it requires a supervisor and aims to augment a client's ability to perform and conform to a directed movement as an explicit goal. The suite of movement tasks also proved to elicit another function, however, in endowing the client to achieve a sense of relaxation throughout the body. Relaxation techniques are often packaged in treatment plans for psychotherapies such as CBT, ACT, biofeedback training, and others, due to their ability to counter the common physical manifestations of stress (Dusek and Benson, 2009). Taut states of the body in the form of chronic or anxious muscle tension have been shown to be relieved by activating the relaxation response system, which is driven by the hypothalamic-pituitary-adrenal axis, and can decrease sympathetic nervous system activity, heart rate, metabolism, and respiratory rate (Dusek and Benson, 2009). Relaxation has been shown to be cost-efficient, health-promoting, and feasible as a self-care skill, as evidenced in the Relaxation Response Resiliency Program (3RP) that was associated with substantial reductions in healthcare utilization (Stahl et al., 2015).

With an understanding of relaxation as a versatile treatment modality, and familiarity with the ability for Dohsa-hou to confer these benefits, Hiroyuki Ohno (2005) developed a new program that repurposed and reorganized the movement tasks to equip the user to achieve a relaxed state on their own. Ohno et al. (2004) and Ohno (2005) narrowed the repertory of Dohsa-hou to a more parsimonious set that can be easily learned by the client while still maintaining the ability to generate a relaxed response. This bodymind approach was dubbed “Self-Active Relaxation Therapy” (SART), namely for its focus on trainee-generated *active* relaxation over the more *passive* reception of trainer-initiated movement tasks directed by the therapist in Dohsa-hou (Ohno et al., 2004; Konno, 2016). SART emphasizes the ability for the user to iteratively recognize points of tension in the body and perform relaxation-inducing movement tasks “self-actively” in this manner on an as-needed basis, as a matter of practical stress management, self-development, and psychoeducation (Ohno, 2006, 2015; Ki and Ohno, 2008; Konno, 2016). SART has been explored for its utility in clinical cases, stress management programs, applications that encourage empowerment in schools and community centers, and employed with residents experiencing the recovery of disaster-affected areas in northern Japan. However, the applications and research conducted using SART have yet to be critically examined or compared to other body-oriented relaxation methods established in the literature. Thus, this paper comprehensively reviews the research and applications of SART, and situates it among the world repertoire at-large to determine its status and prospects as a bodymind approach to resilience, defined as a health practice that facilitates the self-regulation of interoceptive signals through relaxation.

## Methods

### Data sourcing

A systematic search was conducted in English and Japanese for studies including the term *self-active relaxation therapy* (*shudogata rirakuseishon ryoho*) in the research databases PubMed, MEDLINE, Cochrane Library, PsychINFO, WHO Global Index Medicus, Google Scholar, Web of Science, and CiNii (National Institute of Informatics, Japan). The search yielded 21 total articles written in Japanese, and 2 abstracts from academic conferences written in English. Four books have been written in Japanese on the approach. The abstracts were excluded, and the two most recent books, *Basic SART Techniques with Q*&*A* (Ohno, 2007), and *The Training and Specialization of the Clinical Psychologist: Lessons from Disaster Support* (Ki, 2015a), in addition to the 21 Japanese articles served as the basis for this synopsis. Access to these articles and original documents by the creator of the approach (HO), permission to reproduce published figures, and revisions of this manuscript were provided by the co-authors (HO, KH, and YH), which were translated and summarized by the corresponding author.

Among the 21 published articles, four largely stipulate the method, review previous findings, or propose theoretical explanations for the therapeutic effect and mechanism of relaxation. The procedure and principles of SART were extracted from these articles for explanation, and served as a basis for the comparison to relaxation techniques established in world literature. The remaining 17 publications report original research with SART through experimental investigations or applications. Summaries of these studies can be seen in Table 1. The studies were organized chronologically and examined for the scope and setting of SART application, type of experimental design, outcome measurements, and findings. The corresponding section separates the studies by hierarchy of research design and methods of analysis to discuss their level of rigor for future research utilizing the approach. The present article represents the first peer-reviewed, comprehensive, and critical assessment of SART written in English.

**Table 1:** Summary of studies utilizing SART.

**Study**	**Sample size**	**Training group**	**Intervention**	**Duration**	**Outcome measurements**	**Findings**	**Design**
Ki, 2008	2	Adult male with severe motor and intellectual disabilities; female child with intellectual disabilities	SART movements used at a camp for rehabilitation	4-day workshop	Qualitative reports of the training experience from interviews	Pilot tested the potential of “self-activeness” by translating Dohsa-hou tasks into SART relaxation movements; Qualitative reports of positive attitudes toward the body and ability to perform the simpler SART movements in both cases	Case report
Okuzono and Ki, 2010	28	Mothers with newborn infants	SART as activity for maintaining self-control over stress during the childrearing process	4 sessions over 1 month	Kansai Gakuin Parenting Stress Index (92-item), Profile of Mood States (POMS) and Stress Reactions Scale (13-item; Cronbach alpha = 0.80)	SART was demonstrated as one way to buffer child care support through continuous stress management; SART activity associated with significant changes in “vigor”, “anger-hostility”, and “fatigue” on the POMS, and significant pre-post changes in the “bond with child” factor of the KGPSI (but not “confidence as a parent”)	Uncontrolled pre-post study of changes within subjects
Matsufuji and Ki, 2011	6	Infants with developmental disabilities (5 children with Down's syndrome, 1 child with Prader-Willi syndrome) and their mothers	SART as activity to improve parent-child relationships during the childrearing process	4-6 sessions over 1 month	Behavioral observation of the improvement in bodily movement, grasp of the movement task, and interaction between mothers and children; Interview for changes as result of the training experience for mothers regarding child's behavior	Two themes in the types of change emerged: “recognition of child's self-activeness” and an “improvement in willingness to carry out tasks”; Parental relationships were characterized by improved daily interactions and communication; SART activity was associated with more reflection by mothers about the kinds of everyday tasks their children can understand and perform well	Uncontrolled case series of changes within subjects
Murakami and Ki, 2011	4	Elementary school (2 males) and middle school (2 females) children in foster homes	Feasibility study for SART as emotional regulation support skill for children	10 sessions over 3 months	Qualitative reports of the training experience from video-recorded and transcribed interviews	Reported that SART or other body-oriented approaches are feasible for creating client-therapist rapport to discuss daily hassles and offer solutions for adjustment, as well as demonstrated the potential for SART-based programs to provide a continuous support system in the sensitive circumstances of foster care transition for children	Case series report from qualitative analysis of interview data
Utsunomiya and Ohno, 2012	64	Elementary school students (32 in training group class, 32 control group)	Seated version of SART conducted via verbal instruction	2 months	Stress Response Scale for Children (Shimada et al., 1994), and Intrinsic Motivation Scale (Sakurai and Takano, 1985)	No statistically significant change in intrinsic motivation scores in the SART group compared to control, but significant decrease in stress reactivity scores in SART group, especially on the factor for “bodily response”	Case-control study
Yasukouchi and Ki, 2012	5	Children with developmental disabilities (PDD, Asperger's, Down syndrome, intellectual disability, emotional disturbance)	SART training to improve self-esteem in children with developmental disabilities	8 sessions over 4 months	Qualitative reports of the training experience from interviews	Observational reports of lessened rigidity in body movements from guidance and context of SART to raise awareness in the children about the state of their body in daily life, especially at school (i.e., realizing shirt was untucked and taking care of it by themselves); The children were found to have taken more interest in themselves and their bodies, with some implications of improved self-confidence	Case series report from qualitative analysis of interview data
Ishimaru and Ki, 2012	22 119	Mothers with young children	SART as activity for mother empowerment defined as maintaining self-control over stress during the childrearing process	6 sessions over 2 months	Childrearing Anxiety Scale (Aramaki and Muto, 2008), POMS, and Self-Trust Scale (Terasaki, 2009)	Total mood disturbance and tension-anxiety mood states via POMS were found to be reduced as a result of the activity; Mothers qualitatively reported that SART was a helpful tool for them to understand that their stress, irritation, and body states can affect the quality of their interactions with their children, and can adjust their mood through relaxation tasks	Uncontrolled pre-post study of changes within subjects
Ki et al., 2013	11	Special needs elementary school students	Stress management training with 5-min DVD SART	11 weeks	20-item Stress Response Scale for Children and 10-item School Life Questionnaire (Shimada et al., 1994)	Reports of students displaying “good behavior” by faculty instructors monitoring school life improved at post-test; as well as “considerate of feelings when interacting with friends,” “keeps track of time,” and “positivity about moving one's body”; qualitative reports of increased understanding of the movement tasks over time	Uncontrolled case series report of outcomes before, during, and after training
Ki et al., 2014	52 38	Residents of all ages	Disaster support program with SART for tsunami-affected communities in Iwate, Japan (2012-2013)	2 weeks per year for 3 years	GHQ-12 and MHLW Questionnaire of Risk of Disuse Syndrome from Disaster	Focused on repeat users of the Iwate disaster support program and performed ANOVAs for mean change in GHQ-12 and MHLW scale from SART; MHLW functional status results were significant across participants, but GHQ-12 results were nonsignificant; lower psychological distress and higher functional status summary scores were associated with repeat SART program participants as compared to one-time participants	Uncontrolled pre-post study of changes within subjects
Ki, 2015a	1523	Residents of all ages	Disaster support program with SART for tsunami-affected communities in Iwate, Japan (2011-2014)	2 weeks per year for 3 years	GHQ-12 and MHLW Questionnaire for Risk of Disuse Syndrome from the Disaster	Mean changes in GHQ-12 from SART measured by *t*-tests were nonsignificant across participants, but significant for mean changes in functional status measured by MHLW	Uncontrolled pre-post within-subjects implementation
Ohno, 2015	1	Female professional artist in early 50s	Clinical tool for psychoeducation of self-regulation skills	10 sessions over 6 months	Comparison of progress in clinical context from interview	First three sessions the client practiced and performed SART at home, and by the sixth session the client conducted the technique up to twice per day; implemented SART into daily routine to overcome negative mood state and pursue desired goals	Uncontrolled n-of-1 observational study with effect
Ki et al., 2016	173	Junior high school students (74 first-years and 99 second-years) of an all-girls school	SART to improve self-control and self-esteem in a class of 30 second-years students serving as the training group compared to 69 as control; 74 first-years used for grade comparison only	9 sessions over 3 weeks	Junior High School Student Stress Scale, Rosenberg Self-Esteem Scale, Self-Control Scale, and Body Movement and Sensation Questionnaire	Significant post-test mean changes (decreases) in stress reactions, (increases) in self-esteem and self-control for SART training group compared to control group; SART posited as positive and practical life skill for everyday stress management, preliminary evidence of SART use for child self-esteem, stress reactions, and self-control	Case-control Study
Mukasa and Ohno, 2016	8	1 male, 7 female graduate school students	The effect of SART measured via EMG and POMS	3 months and 2 weeks	POMS and structured 15-min interview	Muscle activity change confirmed via EMG; SART was associated with reduced total mood disturbance (TMD) and tension-anxiety on POMS, and a qualitative analysis of interview data revealed numerous categories related to changes in body awareness after experiencing the approach	Experimental study; Qualitative analysis of interview data
Takeo et al., 2016	8	Elderly residents of areas affected by disaster	SART as activity to build rapport, discuss, and understand the impact of the disaster experience	4 days	Text analysis of remarks that were categorized, labeled, and allocated by time perspective	Content for 3 of the 8 interviews thoroughly analyzed using the Modified Grounded Theory Approach (MGTA); Numerous themes related to the past and recollections of the time of the disaster reflecting the continuous impact on their lives; Investigator reported that it was easier to discuss these difficult experiences upon undergoing SART with the trainer	Qualitative study with interview data
Ki et al., 2017	2 12	University students (6 assigned to single-SART group, 6 to therapist-assisted SART group)	The effect of therapist-assisted and SART practiced by oneself measured via electromyography (EMG) and questionnaires in 6 sessions to determine which version is more likely to be adhered to after training	5 sessions over 2 months	EMG, Sense of Movement Scale (SMS), Emotional Experience Scale (EES), Task Comprehension Scale	EMG-measured change from SART arm raise relaxation movement at post-assessment confirmed compared to an EMG control movement task; Analyses of change conducted via *t*-tests for all scales between both groups; therapist-assisted group scored higher on items related to “feeling of bodily change” and “sense of control” on SMS, “pleasantness” and “initiative” on EES, and “attitude toward the movement task” on the TCS; The single-SART group experienced trial-and-error while performing the movement tasks on their own, but by the fifth session adjusted, and the factor for “feeling of relaxation” in the single-SART group increased	Experimental Study; Pre-post study of changes within subjects between therapist-assisted SART and autonomous SART
Mori and Ohno, 2017	3	1 female in her 30s with PDD/OCD/possible ADHD, 1 male with Asperger's syndrome in his 20s, and another male in his 30s	SART as body-mediated communication	10 sessions over 5 months	Semi-structured interviews	Through analysis of post-activity interviews, SART was found to facilitate communication related to self-understanding and positive change related to daily life goals in the three cases	Case series and qualitative study of interview data
Motomura and Ohno, 2017	48	Mothers of children with either pervasive developmental disabilities (PDD) or attention deficit/hyperactivity disorder (ADHD)	SART as activity for stress management during childrearing process	2 months	Stress Self-Rating Scale, Checklist for Disabilities Scale in Young Children, and POMS	Mean decreases at post-test on every rating of cognitively appraised stress response for all mothers regardless of nature of their child's disability except for “energy level”; Free comments about the SART activity revealed “fatigue” to be major factor in their daily experience of childrearing	Uncontrolled pre-post comparison of changes within subjects

## Results

### The procedure and principles of self-active relaxation therapy

Ohno (2007) and Ki (2015b) explain that SART is designed to stretch, twist, and release areas of the body through a series of movements which are guided by the practitioner and performed by the client. In the intake process, the therapist begins by asking the client if they have any particularly tense, sensitive, or painful areas in the body that should be avoided out of safety or other considerations. The program takes ~24 min to perform, and begins with a pre-assessment where the client sits cross-legged with their arms extended and hands placed together in front of them (Supplementary Figure 1). The client moves their outstretched arms to the right as far as they can while making a mental note of how far they could do so comfortably and maintain the position, then returns to the center and repeats the same action on their left side. The client then conforms to a lateral recumbent position, which serves as the basic posture for SART (Supplementary Figure 2), usually with the knee brought forward for support of a neutral spine. SART was conceived using this form, but a 5-min seated version typically instructed via DVD was developed for clients in wheelchairs as well as for shorter salon activities. The figures detailing the procedure in this review indicate the cooperative version of the program that is conducted between the therapist and the client for the purposes of introduction, but there are slight modifications for doing the movements completely by oneself (available upon request). The degree of relaxation is described as a function of time spent doing the relaxation tasks, with the full 24-min program generating a moderate sense of relaxation, and the 5-min version granting a milder subjective sense of relaxation (Ohno, 2015).

In Ohno (2007), each group of movement tasks is defined as a “system” that corresponds to the section of the body that each set of movements is meant to target. System 1 is comprised of movements to relax areas in the upper body via abduction of the shoulder (above the torso; Appendix 1), System 2 targets the lower body with abduction of the hip (below the torso; Appendix 2), and System 3 invokes a multi-sectional stretch across the body, with the trainer intervening to create a deeper stretch (Appendix 3). Each movement is performed at least 2–3 times per side of the lateral recumbent position, and all movements are done on both sides (left and right). The first attempt familiarizes the client with the goal of the movement, the second attempt provides the therapist with the opportunity to carefully correct the degree, direction, or intensity of the movement, and the third attempt functions to solidify the client's kinesthetic sense of the goal of the movement as a matter of psychoeducation and autonomous future practice. Basic nomenclature and short descriptions for the movements of the three systems accompany the figures in Appendices 1–3 (Ohno, 2005, 2007).

Ohno (2007, 2015) stipulated the psychoeducational purpose and theoretical underpinnings of SART as learning the sets of movements with a representation of body symmetry and positively accepting the immediately felt sense of self as it currently exists in the “here-and-now.” Becoming viscerally aware of the state of one's bodily condition is described as a major goal of the movement tasks. Ki and Ohno (2008) explain that this was based on clinical observations of Dohsa-hou practice to which it was found that when movements are enacted on one's own, the “experiencing process” of synchronizing the intention, initiation, and effort to move and relax the body regulates attention to the present moment of sensation (Ki and Ohno, 2008; Ohno, 2015). This embodied coherence ideally allows the user to witness a change within themselves and re-assert a sense of control over their bodily state due to the experience of dissipated tension and the calming effect of relaxation (Ohno, 2015).

Ohno (2015) emphasizes that one the most important tenets of the approach is that the client in question decides for themselves the most stable position through iterative adjustments derived from communication with the therapist or practitioner. The main role of the practitioner is to guide the movements with verbal cues, support the negotiated stable position and monitor the maintenance of a neutral spine. This rapport is realized through encouragement from the trainer to the trainee when they perform movements smoothly and by challenging the user to push beyond the initial limit of their range of motion where feasible (Ohno, 2015).

### Case reports and qualitative analysis of interview data

A few studies have employed qualitative analyses of interview data to learn about the nature of the therapeutic effect of SART, as well as whether body movements performed to achieve relaxation can be usefully applied to enhance body or self-awareness in the daily lives of two groups: individuals with developmental disabilities and elderly residents of disaster-affected areas in northern Japan.

Ki and Ohno (2008) discovered that SART was useful as a support activity at a 4-day psychological rehabilitation camp for two individuals with special needs: an adult male with severe motor and intellectual disabilities and a male child with intellectual disabilities. The case report pilot tested the potential of Dohsa-hou movements based in “self-activeness” which evolved into the relaxation movements of the SART program. Interviews about the training experience showed that both individuals could grasp and perform the simpler movements, and reported positive attitudes toward their bodies over the course of the workshop. This preliminary set of findings was the result of a short-term observation but affirmed a proof-of-concept for use with actual clients with special needs. As SART was shown to appeal to bodily change under the more constrained circumstances of communication associated with those managing developmental disabilities, this shows that it could be usefully transferable to neurotypical contexts. However, the lack of control conditions, randomization, statistical analysis, or tractable outcome measurements that attenuate variables makes it difficult to determine the degree to which changes were amenable to SART.

Takeo et al. (2016) interviewed eight elderly residents affected by Japan's disasters of March 11, 2011, after undergoing SART over the course of 4 days. The approach was investigated as a salon activity to provide a context to discuss the impact of the disaster experience. Audio of the interviews was recorded with permission and transcribed, and three of the eight interviews were selected for analysis using the Modified Grounded Theory Approach (M-GTA). The transcriptions were aggregated in accordance with their major thematic content and ordered as a process over time. Many comments by interviewees were found to be concerned with the past and present, and the investigators surmised that this might reflect a continuous impact of the disaster on their lives. With regard to the SART activity, the elderly residents' self-reported free comments mentioned that it made them feel “relaxed and at-ease,” “able to sleep better,” and “able to more smoothly conduct daily activities” at the final interview. The principal investigator concluded from the context of these findings that it was easier to discuss difficult experiences of disaster-related adversity upon conducting SART with the disaster support volunteer.

This qualitative analysis shows some small support for SART as an “icebreaker” activity for building trust between clients and therapists, or disaster support volunteers and elderly residents of affected areas. However, it is unclear how the interviews were chosen for M-GTA analysis, and if the thematic emergence of the past and present was due to the choice of questions in the structured interview or from an extemporaneous focus of discussion at the time of the interview. It is also unclear if the reports of positive psychological change are attributable to the activity or possibly confounding individual differences. The extent to which SART was responsible for their reports of daily life changes and the duration of these changes over time is unclear from the lack of a follow-up study. There are numerous diagrams of process analysis in this study that serve to theorize about mechanisms of time perspective, suggesting that this study was less concerned with the effect of SART and more about the nature of the processing of the disaster experience in general. Therefore, this study represents one of preliminary observation and mechanism-based reasoning associated with a lower grade of evidence.

### Case series and feasibility reports with interview data

As SART grew out of a clinical psychology application of relaxation tasks, a number of studies have undertaken a case series observational design with clinical interview data collection and analysis. Although the reported sample sizes of the studies in this section are extremely small, some aspects of the applicability of SART beyond the clinic and into support group community psychology contexts were tested in the following experiments while incorporating the same clinical interview research methodology.

Mori and Ohno (2017) qualitatively investigated SART as a tool for facilitating self-awareness and daily life goals with body-mediated communication in a 10-session, 5-month case series of three individuals with developmental disabilities: a female in her thirties with pervasive developmental disorder (PDD), OCD, and possibly ADHD, and two males with Asperger's syndrome in their twenties and thirties. Semi-structured interviews during each session were conducted to investigate the process of change in attitudes about the self and the body over time, with SART used as a clinical tool and case reports of client feedback as an evaluation. Two of the three cases revealed initially negative attitudes toward the self, with one client reporting difficulties controlling his body and only expressing a vague sense of self-awareness. The investigators observed improvement in the expression of mood and bodily states about the self over the course of the sessions, and concluded that communication via body movement was increasingly facilitated in the three cases over time, with SART allowing one client to bring attention to their bodily sensations halfway through the total number of sessions and performing it daily by the final one. This longitudinal clinical case series over 5 months of process-based change in body and self-awareness demonstrated feasibility and clinical utility by showing that SART could be learned and utilized with adult clients with developmental conditions. While the longer-term qualitative findings of Mori and Ohno (2017) extend upon the short-term workshop use of the approach in the context of developmental disabilities in Ki and Ohno (2008), the small number of cases, lack of control conditions, randomization, or analysis of measurable outcomes with psychometric instruments makes it difficult to extrapolate the effect of SART beyond a relatively modest claim of clinical significance.

Murakami and Ki (2011) conducted a study to determine if SART can feasibly endow or otherwise enhance emotional regulation skills in two male elementary school students and two female middle school students facing the transition and living circumstances of foster care. The children were met for 10 sessions over the course of 3 months, and the post-SART training interviews were video-recorded and transcribed for case series comparison. The context was reportedly useful for discussing daily issues and offering solutions for adjusting to living in a house with many people. The strength of this study lies in its affirmation that SART could serve as a supplementary tool in a continuous support system for children in foster care, especially insofar as children could interface with daily hassles and therapists could promote skills for emotional regulation. However, the weaknesses include a lack of detailed reports from the children about their ability to obtain these skills via SART and precisely which sensitive aspects of the foster care experience are important to address with body-oriented approaches. Adverse effects attributable to this application of SART, if any occurred, were not mentioned.

Matsufuji and Ki (2011) devised a program with SART as an activity for mothers to feel closer with their infants with developmental disabilities during the childrearing process. The infants (five with Down's syndrome, one child with Prader-Willi syndrome) ranged from 2 months to 19 months old. Behavioral interactions between mothers and infants were observed. Mothers were interviewed about their child's behavior over the course of 1 month to compare changes in the parent-child relationship as a result of the training experience. Two themes in the types of change emerged from analysis of the interview data: an increased “recognition of child's self-activeness” and “improvement in willingness to carry out tasks” as witnessed by mothers. The key finding was that SART activity was associated with more reflection by mothers about the kinds of everyday tasks their children can understand and perform well. The main limitations were a lack of specific variables related to childrearing stress or anxiety to be measured and unclear criteria for demonstrating an improved parent-child relationship beyond theoretical considerations.

Yasukouchi and Ki (2012) explored SART as a tool to improve self-esteem in five children with developmental disabilities (PDD, Asperger's, Down's syndrome, intellectual impairment, and emotional disturbance, respectively) through qualitative reports of the training experience from interviews of the children and their parents in a case series lasting eight sessions across 4 months. Parents observed that their children's body rigidity seemed to lessen over time and reported that SART appeared to raise awareness in the children about the state of their body in daily life, especially at school. This study demonstrates the feasibility of SART over a long-term observation of process-based change in a known group, but it is limited by its small sample size, lack of randomization, lack of control group, and unclear criteria for interpreting successful outcomes for children with developmental disabilities. The qualitative analysis and behavioral observation design lack the specificity to parse the training effect of SART from the context of increased attention to issues of body awareness and control.

### Uncontrolled observational study with effect in a clinical context

Clinical applications of SART have been numerous in Japan, but one case illustration of a client presenting with alcohol dependency and depressive symptoms was described as an exemplar in the literature (Ohno, 2015). A 50-year-old female professional painter presented with the claim that no inspiring images worth painting had appeared in her mind for around 9 years, and that she wanted to be able to paint passionately again. One-hour clinical interviews with SART were conducted once a month for a total of 12 sessions. The therapist assisted the client in performing the full program of movement tasks from all three systems. After the first three sessions, the client began to practice and conduct the relaxation tasks at home. The client reported that when she felt that it was hard to get up in the morning and experienced depressive symptoms, she performed SART on her bed. By the sixth session, the client stated that they had begun a rhythm of performing the relaxation tasks on their own up to twice a day. The client began to integrate other activities into this routine incrementally, such as rearranging their room to be able to paint and even working on a new self-portrait. At follow-up from the last session, the client reported feeling more active and productive, and informed that she would soon open an exhibition for her new artwork (Ohno, 2015).

This case demonstrated a versatile, process-scaffolding purpose of SART as both a clinical tool for the therapist and a self-care skill for the client, which is based in body awareness and actionable as a self-development platform to regulate mood, pursue valued goals, and commit to desired activities. Adverse effects attributable to SART, if any, were not reported. While the time course from the clinical interview detailed the point at which a user might implement the technique on their own, the lack of outcome measurements leaves the magnitude of the therapeutic effect afforded by SART for depressive mood unclear. Although it was reported that the client began to undergo the technique up to twice per day, the assessment of home practice of the SART intervention was also unspecified. Reliable psychometric instruments for each session and relaxation worksheet homework for tracking at-home practice could help capture changes more directly and determine the effect of the SART with greater precision.

### Uncontrolled pre-post experiments with outcome measurements

Other experiments with SART measured outcomes through psychometric instruments taken before and after learning the relaxation technique to measure changes in participants from three research populations: mothers, special needs students, and residents of tsunami-affected areas in Japan.

Okuzono and Ki (2010) used SART as a way to buffer child care support through continuous stress management for 28 mothers (*M* age = 36.52 years old, *SD* = 4.5 years old) with newborn infants. This study operationalized the Japanese version of the Kansai Gakuin Parenting Stress Index (KGPSI; Abidin, 1983; Nozawa, 1989), Stress Reactions Scale (Niina et al., 1990), and the Profile of Mood States (POMS; Lorr et al., 1982; Japanese version by Yokoyama et al., 1990). SART activity was associated with significant changes in “vigor,” “anger-hostility,” and “fatigue” on the POMS. Significant changes were also found for the KGPSI factor of “bond with child,” but SART notably did not elicit pre-post changes in “confidence as a parent”. Mothers expressed a strong desire to continue the program on a questionnaire evaluation, but a follow-up study was not performed. The modest sample size, lack of objective measurement, need for stronger links to mechanisms of stress and anxiety or the psychological needs of mothers were acknowledged as limitations. Without a control group, it is unclear if the findings of positive change were due to SART itself or the context for support provided by the program.

Ishimaru and Ki (2012) conducted two studies with 119 mothers (*M* age = 35.0 years old) of young children to investigate SART as an empowering mood regulation skill. The mothers underwent SART with their children for six sessions across the span of 2 months. The Childrearing Anxiety Scale (Aramaki and Muto, 2008), Feelings of Self-Trust Scale (Terasaki, 2009), and POMS were measured for changes in a pre-post training design. Comparison of the first and sixth session scores showed that all mood states measured via POMS, except for “vigor” but especially total mood disturbance and “tension-anxiety,” were found to be significantly reduced as a result of the activity, but notably lacked an effect for the other scales. Mothers qualitatively reported that SART was a helpful tool for them to understand that their stress, irritation, and body states can affect the quality of their interactions with their children, and that by adjusting their mood through relaxation tasks, they can stay cognizant of them in everyday childrearing situations. However, SART did not tap the dimensions of the Feelings of Self-Trust Scale, notably showing no effect for self-affirmation despite the iteratively encouraging feedback that occurs between the client and therapist, and suggesting that the SART effect is more reflexive rather than volitionally attending. The main limitation is the difficulty in separating the SART effect on mood regulation skill enhancement from the benefits of group interaction that the mothers and children may have experienced through increased contact and attention.

Ki et al. (2013) packaged SART relaxation tasks in a stress management program application for 11 special needs students over the course of 11 weeks. They conducted stress management training using the 5-min DVD of seated SART and measured changes via the 20-item Stress Response Scale for Children (SRS-C) for students and the 10-item School Life Questionnaire for on-site faculty (SLQ; Shimada et al., 1994) in a pre-post training design at three time points (first, sixth, and eleventh week). A significant decrease in the average stress score was observed at the final assessment. Faculty instructors reported that students displayed a significant improvement in “good behavior” on the SRS-C, as well as improvements on items related to “consideration for others,” “getting along with friends,” “keeping track of time,” and “positive attitude toward their body” at post-test. This study demonstrated the utility of the short version of SART as a stress management tool in special needs education. However, the modest sample size and lack of control group make it difficult to separate the relaxation effect from the opportunity for support.

Mukasa and Ohno (2016) found that anxious physiological states can be changed in a n-of-1 experimental study and in a case series lasting 14 weeks. One male graduate student underwent EMG measurements as a proof-of-concept that muscle activity change could be seen in a seated SART movement of shoulder abduction as compared to a neutral maintenance task, and the effect of SART on muscle activity change was confirmed. POMS and a structured 15-min interview were conducted for separate group of seven female graduate students. SART was associated with reduced total mood disturbance and tension-anxiety, and a qualitative analysis of interview data revealed numerous categories related to changes in body awareness after experiencing the approach. This study found changes in mood states within subjects as a baseline, but without a control group or other experimental conditions, it is difficult to parse the role of relaxation from SART as the arbiter of mean decreases.

Ki et al. (2017) compared the differences between the therapist-supported and autonomous at-home versions of the program with university students. A total of 12 participants (six assigned to the therapist-assisted SART group, six in the “single-SART” group) underwent EMG measurements taken at pre- and post-assessment, a full session of all three SART systems, and a questionnaire battery with items from the Sense of Movement Scale (SMS), Emotional Experience Scale (EES), and Task Comprehension Scale (TCS) that was administered before and after five sessions over the course of 2 months. Analyses of change conducted via *t*-tests for all scales were compared between both groups. The therapist-assisted group scored higher on items related to “feeling of bodily change” and “sense of control” on the SMS, “pleasantness” and “initiative” on the EES, and “attitude toward the movement task” on the TCS, whereas the autonomous single-SART group scored higher on the “self-activeness” and “feeling refreshed” items of the EES. Notably, the single-SART group reported that they experienced some trial and error while performing the movement tasks on their own, but by the fifth session gradually adjusted, and the factor for “feeling of relaxation” in the single-SART group increased in later sessions. This is an important distinction for the difference between the two SART modalities and the process inherent to cultivating it as a habit. While this study controlled for two training group types, it did not include a non-training group as a baseline, and the assessment of home practice by the single-SART group was not tracked as homework but reported in subsequent sessions, leaving the self-regulation aspect unclear.

Motomura and Ohno (2017) examined a SART-based stress management modality for 48 mothers of children with either pervasive developmental or attention deficit disorders. The Stress Self-Rating Scale, Checklist for Developmental Disabilities in Young Children, POMS, and semi-structured interviews were administered to mothers whose stress reactions were compared by separation into groups of perceived high and low severity of their child's developmental condition. Effects from SART in the form of stress response reduction and cognitive appraisal change were observed across all groups regardless of the severity of the child's disability. Analysis of the interview data revealed that “childrearing” and “household chores” mutually influence each other and contribute to fatigue in the sample of mothers. The authors surmised that reducing the stress response “through one's own power” might have led to the change in cognitive evaluation, and might represent the vehicle of stress management conferred by SART more broadly. The degree that SART-based relaxation contributed to the within-subject changes has some support from the results, but the lack of control group makes it difficult to parse the therapeutic effect of SART as a tool from the psychoeducational context surrounding the activity.

### Case-control studies with outcome measurements

Two studies incorporated a case-control design with comparisons of changes in summary scores on psychometric tests between groups to observe the outcome efficacy of SART.

Utsunomiya and Ohno (2012) investigated the 5-min shortened version of SART as a tool for stress management for elementary school students. Two classes were used at a Japanese elementary school with one class treated as a control group. A total of 62 students (26 males, 26 females) were surveyed for stress responses via the SRS-C (Shimada et al., 1994), and the Intrinsic Motivation Scale (Sakurai and Takano, 1985). SART showed a significant effect of reducing “depressive” and “anxious” feelings, and “bodily responses” as a result of the program, but notably showed no effect on intrinsic motivation. This study is one of the few with the benefit of a control group, but the school site selection appears to have been determined by convenience and randomization was not reported.

Ki et al. (2016) attempted a stress management application of the same 5-min SART to tap self-esteem and self-control in a sample of 173 children, comprised of 74 first-year and 99 second-year junior high school students Ki et al. (2016). One class of 30 second-year students was designated as the SART training group, and the remaining 69 second-year students served as the control group. The Junior High School Student Stress Scale (Yamamoto and Hukui, 2009), Rosenberg Self-Esteem Scale for Elementary Students (Susaki and Anii, 2013), Self-Control Questionnaire (2006), and SMS (2007) were employed as psychometric instruments in a pre-post activity design in which students conducted SART after their lunch period for three sessions per week over the course of 3 weeks. The 74 first-year students filled in the same questionnaires to compare scores between grade levels, but did not contain a training group. The students in the SART group reported that they enjoyed the activity and felt refreshed and energetic, and the training was significantly associated at post-test with lower scores in anger and anxiety, fewer stress reactions, and higher self-esteem and self-control scores as compared to the control group. This study provided preliminary evidence for a relationship between SART use and self-reported self-esteem, self-reactions, and self-control in the shorter version associated with milder relaxation, but the process of determining which class would serve as the training group was not reported and the status of systematic assignment remains unclear.

### Study of large-scale program implementation in a disaster mental health setting

The application of SART of greatest interest and investment was its utilization by Ki (2015b) in communities of Miyako City, Iwate Prefecture, Japan, that were directly affected by the Great East Japan Earthquake and Tsunami of 2011 (Ki et al., 2014; Ki, 2015b). This program identified areas in need with bottom-up coordination of local stakeholders and door-to-door recruitment at temporary housing complexes to provide a mental health promotion service for the communities. Locations of implementation were largely meeting rooms of prefabricated temporary housing complexes, city hall rental rooms, and NGO-related facilities. The program was provided twice a year, consistently available in the third week of March and first few weeks of August, for the past 6 years since the disaster.

As reported in Ki (2015b), the grand sum of residents of the area that have undergone the program totaled 1,523 (2011–2015) individuals. The psychometric instruments used to test the efficacy of the program for its major clientele of elderly residents were the 12-item General Health Questionnaire for psychological distress and the Japanese Ministry of Health, Labor and Welfare (MHLW) Risk of Disuse Syndrome from the Disaster Questionnaire for measuring changes in functional status or physical inactivity since the disaster (Ki et al., 2014; Ki, 2015b). In published findings of four measured time points of the community application, repeated measures tests for the analysis of variance showed a significant difference for functional status via MHLW, but did not show a significant difference within groups for GHQ-12 as a result of SART practice (Ki, 2015a). Overall, residents of disaster-affected areas in the early stages of recovery qualitatively reported some instances of intrusive thoughts about the disaster, trouble sleeping, and repetitive thoughts about their tenuous state of residence and the security of their pension, but that the SART program experience gave them a short-term outlet for making their body feel “looser and lighter” with boosts in energy and “vigor” as a result of the activity (Ki et al., 2014).

The relatively steady number of individuals assisted per administration and numerous qualitative reports of positive change suggest that SART as a program has fostered mental health promotion and served an important role among health services in the tsunami recovery context. As the majority of clientele were known to be elderly residents, the scales operationalized in the application appear to have been thoughtfully chosen. While it was reported that residents who were repeat participants of the program continued to use the instructive flyer with figures of the SART tasks and desired the instructional DVD for use at home, there was no formal assessment of the home practice of the intervention (Ki et al., 2014; Ki, 2015b). The lack of a significant effect for users of the program on self-reported GHQ-12 illustrates a notable limitation for the effect of SART to produce a change in broadly defined psychological distress, although repeat users of the program were found to score lower on the GHQ-12 scale of psychological distress and report fewer issues on the MHLW questionnaire generally (Ki et al., 2014; Ki, 2015b).

The study contains ample sample size, outcome measurements, a longitudinal design that provides follow-up opportunities, and a quasi-randomized sampling recruitment, but not random assignment, of participants as affected residents chose to undergo the activity on their own accord at temporary housing meeting rooms chosen based on expressed need from community centers and logistical considerations determined by the coordinators. Reports of continued use notwithstanding, the lack of home assessment leaves the degree of self-regulation provided by SART practice alone unclear. Individual differences in the response to relaxation were not measured. In addition, the MHLW scale notably has no English equivalent, and it is unclear if its psychometric properties have been verified. It is also possible that the consistent number of repeat participants has been due to the general mental health promotion services of social support that have been provided over the motivation to relax via SART.

## Descriptive comparison to methods established in the literature

### Body movement basis: yoga poses and the alexander technique

Yoga uses body movements to develop core strength, improve muscle flexibility, and challenge the user to undergo self-development by physically conforming to poses designed to overcome perceived limitations. SART is considerably less sophisticated than yoga in terms of targeted functional anatomy, but shares some of the movement elements for supine poses. A clear overlap appears to be the basic supine twist (*jathara parivrtti*) with leg extension in yoga, which stretches the *pectoralis major*, external and internal obliques, *gluteus maximus*, and hamstrings (Kaminoff et al., 2007). SART movement elements from the System 2 lower body trunk rotation at the hip joint and the System 3 simultaneous trunk rotation and horizontal abduction of the shoulder have similar targets in functional anatomy. However, the general locus of the lateral recumbent position as the basic posture for SART appears to be a departure from conventional yoga poses which encompass more diverse and complex positions such as standing, kneeling, sitting, prone, and others. The yoga approach to using gravity to work muscles and the focus on dissipating excess muscle tension in the lumbar spine also appear to cohere to the movement elements and goals of the SART program. SART, however, is less focused on the exercise and introspective elements found in forms of yoga.

In addition, SART may follow a self-regulatory mechanism of top-down control of bottom-up interoceptive signals akin to the model of improvements in reactions to the stress response that are proposed to occur in the practice of yoga (Gard et al., 2014). However, the therapeutic effect of SART is hypothesized to be more reflexive and less volitionally attending as yoga or as contemplative as practices like mindfulness, as it has been shown to provide a more state-based, instantaneous means of relaxation that targets mood, motivation, and energy (Ohno, 2007, 2015). The relative lack of deep meditative stance while undergoing the SART suite of movement tasks would also seem to separate it from tai chi, qigong, hatha yoga, and other forms of meditative movement.

The Alexander Technique has typically been applied as a body-oriented approach to postural improvement, performance readiness, and chronic pain management (Little et al., 2008). The Alexander Technique emphasizes care with movements of the head, neck, and back to lessen strains on the body that could lead to compression and teaches the client principles to improve breathing capacity (Woodman and Moore, 2012). The therapist brings attention to unconscious movement habits within the client and rehearses them with iterative feedback from chair and table work. Success in performing the Alexander Technique seems to rely on a strong and sustained sense of awareness of the body at any given moment, especially when applied to performance. This is beyond the scope of the goals of SART in which such an acute attenuation of body awareness is comparatively brief and experienced as an appraisal of the subjective sense of relaxation that SART provides. On the surface, the standing exercises of Dohsa-hou appear to resemble the Alexander Technique more than SART. Dohsa-hou directly targets postural stability and a heightened sensitivity to the role of body movement in everyday life (Konno, 1999). SART emphasizes postural assessments less than the experience of range of motion change and the achievement of a client-derived sense of moderate relaxation (Ohno, 2015).

### Guided relaxation: progressive muscle relaxation, autogenic training, and breath therapy

Progressive muscle relaxation (PMR) was developed by Jacobson in 1929 and continues to be one of the most prominent relaxation induction methods in the literature. In classical PMR, the therapist guides the client to flex and release a series of muscle groups, typically starting from the lower body upward and holding the contraction for up to 5 s per muscle group (Vancampfort et al., 2013). According to Ohno (2010) and Ki and Ohno (2006), PMR was a point of reference for the development of SART. The guided relaxation aspect of PMR occurs in SART, but differs in that the effects on the musculature are self-derived via contraction while still rather than by static stretching while supine (Ki and Ohno, 2006).

Autogenic training (AT) was created by Schultz (1932) and shares some aspects of the SART-based approach insofar as it is a method that facilitates autonomic self-regulation. AT uses self-guided image-based instructions that induce a state of self-hypnotic relaxation to reduce arousal, which has been usefully applied as a stress management technique for lessening tension, stress and anxiety in clinical contexts (Linden, 1994). SART, however, does not incorporate this kind of explicit imagery or suggestion at any point during the performance of the movement tasks or the pre- and post-assessments.

Breathwork in body psychotherapy incorporates attention to the breath as a source of guidance for relaxation from the therapist (Caldwell and Victoria, 2011). Breath therapy integrates many bodymind approaches with coping skill implications and improvement in body insight for managing chronic pain (Mehling et al., 2005). These methods operationalize posture and emphasize chronic lower back pain management in a manner that bears resemblance to the therapist-initiated directions and applications of Dohsa-hou. SART does not provide guided movement coordination with breath to this degree, and only uses breath to physically allow for increased range of motion and deepened stretches on a select few of the movements (e.g., to open the chest more effectively in horizontal shoulder abduction). Thus, the instructions do not appear to reach the level of guided breathing as an explicit goal typically found in breath therapy, yoga, or mindfulness meditation (de Jong et al., 2016).

### Creating rapport for regulating mood via the felt sense: focusing

Focusing was developed as an attention regulation technique that simultaneously sets the groundwork for the client and therapist to develop trust in the psychotherapeutic process. As described in (Gendlin, 1991), connections between focusing and SART may be seen in the attempt to attenuate sensations to the here-and-now, as well as in the process of fostering a trusting therapist-client relationship through a safe environment. Focusing in the Gendlin tradition specifically targets stuck points or “dead-ends” in therapeutic problem-solving. It is characterized by drawing attention to inner experience, or the felt sense, which on the surface seems to be one of the closest interpretations of the Japanese word used in SART and Dohsa-hou known as *jikkan*, or the “realization of actual sensation.” In addition, its roots in Rogerian psychotherapeutic principles where the client is placed at the center and given the tools to drive positive change resembles the “self-active” tenet of SART (Ohno, 2007). Overall, the mechanism of focusing may be similar to the aspect of SART that recalibrates the locus of attention to the present moment of experience via instantaneous body awareness. However, focusing and focus-oriented psychotherapy appear to invoke more volitionally-attending concentration than SART, that is, the therapeutic experience during SART is considerably less contemplative than focusing.

### Psychoeducation as foundation: the feldenkrais method

The Feldenkrais Method is characterized by enhancing and discriminating types of body awareness to exchange potentially maladaptive habitual patterns of movement (Buchanan and Ulrich, 2001). Similar to SART, it has been shown to contribute to reductions in negative mood states as measured by POMS (Kolt and McConville, 2000). SART shares some of the same goals for psychoeducation and self-development as the “somatic education” inherent to the Feldenkrais Method. This is especially the case for forming a habitual practice with SART as a tool for self-regulation of mood. However, SART is less focused on conforming to explicit body configurations as therapist-directed tasks for direct postural or “motor behavior” outcomes, and more on empowering the client to learn to perform the tasks by themselves when they deem that the need arises. The postural aspects of the Feldenkrais Method, in this light, appear to adhere more to the program goals of Dohsa-hou.

### Disambiguation from dohsa-hou

Ohno et al. (2004) stipulated SART from the repertoire of existing Dohsa-hou movement tasks, thereby sharing its lineage as an approach. Ki and Ohno (2008) observed the role of self-activeness in the Dohsa-hou tasks which began a line of derivation, but sharp distinctions between SART and Dohsa-hou have seldom been thoroughly delineated. However, Konno (2016) recently reviewed the historical underpinnings of Dohsa-hou and the methods that have emerged along the lineage, and mentioned that the major difference between Dohsa-hou and SART is the active role of the client and his or her understanding of the movements for future autonomous use. The present review extends the literature on this distinction. Dohsa-hou is comprised of more extensive motor vocabulary that emphasizes conforming to a therapist-initiated movement task, whereas the primary driver for the organization of the three systems in SART is to endow the client with a simple and intuitive method that can be conducted on one's own to manage stress and regulate mood states.

## Discussion

### Key findings

From the preponderance of current literature on the approach developed and explored in Japan, SART appears to be a flexible bodymind practice for inducing relaxation and obtaining the process-scaffolding of “self-active” psychoeducation so that the user can repeat or continue the practice on their own terms (Ohno, 2007, 2015). From this basis, it appears that SART movement tasks fall under the category of *simple instrumental actions* that users can execute daily and be co-opted through relaxation to perceive control and agency over bodily states (Haggard, 2017). The wide range of applications appears to be a major advantage of the approach, namely that the body-oriented focus makes it open to people with a broad scope of health conditions as an activity or intervention. Programs with SART have been developed for clinical settings (Ki and Ohno, 2008; Ohno, 2015), university students (Mukasa and Ohno, 2016; Ki et al., 2017), schoolchildren (Utsunomiya and Ohno, 2012; Ki et al., 2016), children in foster care (Murakami and Ki, 2011), special needs children (Yasukouchi and Ki, 2012), parents (Matsufuji and Ki, 2011; Ishimaru and Ki, 2012; Motomura and Ohno, 2017) the elderly (Takeo et al., 2016), the physically handicapped (Ki, 2008), and residents facing disaster recovery (Ki et al., 2014; Ki, 2015a). It has been helpful for creating a context to engage in reflective listening, rapport, and stress management (Ki, 2015b). The method of instruction between client and practitioner is designed in such a way that a given person's intuitions about body symmetries can be leveraged to understand, attempt, and remember the suite of movement tasks (Ohno, 2015). The relatively quick, apparent, and actual sensation of mood state from bodily change endowed by SART practice also allows the therapist to interpret the client's state of tension as expressed through their body, which may open a new avenue of communication that might be masked in the conventional talk-based psychotherapeutic experience. In this manner, SART allows for a narrative, one-on-one client focus to personalize the psychotherapeutic experience by navigating and negotiating set points of a person's body tension and discussing changes in bodily states.

Self-regulatory skills inherent to the deliberate practice of meditation, yoga, tai chi and other approaches have been tied to the experience of a positive upward spiral (Garland et al., 2015), affect labeling and reappraisal (Burklund et al., 2014), functional autonomy (Gonçalves et al., 2011), and other holistic components of adaptive health competence. Many of these approaches were promoted in the third wave boom of psychotherapies and have been recently advocated as alternatives to talk therapies and drugs in groundbreaking research on resilience and traumatic stress (Van der Kolk, 2014). Some researchers have integrated such methods into an overarching toolbox of meditative movement (Payne et al., 2017), but SART appears to be aligned with the reflexive rather than volitionally attending aspects of these techniques as it does not entail the explicit introspection or concentration found in meditation (Payne and Crane-Godreau, 2015). SART shares the body movement emphasis of yoga and the Alexander Technique, the guided process to relaxation and muscle contraction of PMR, the psychoeducation aims of the Feldenkrais Method, and the here-and-now aspect of focusing. Notably, the contrasts between SART and relaxation methods in world literature reported in this review are limited by the fact that empirically derived effect size comparisons with studies implementing controlled designs have yet to be conducted. However, on the basis of the characteristics, goals, and implementation procedures of the approaches, Dohsa-hou appears to adhere more closely to the techniques in world literature designed to change unconscious movement habits and enhance body consciousness. Despite growing out of the same motor vocabulary, the active, client-generated aspect SART differs from the more passive, therapist-driven approach of Dohsa-hou (Ki, 2008; Konno, 2016). SART appears to be a bodymind practice that acts on body sensations in a way that restores a person's sense of presence in the world by matching the motor prediction of simple instrumental actions with actual body feedback and outcomes, leveraging movement to generate an awareness of sensation by modulating our *feeling state* to accept or reframe interoceptive signals in the present moment (Paulus and Stein, 2010; Calì et al., 2015; Farb et al., 2015; Asai et al., 2016).

### Limitations based on study methodology

The majority of research studies regarding SART have been uncontrolled before and after within-subject designs with relatively modest sample sizes, chiefly investigating application efficacy, feasibility, transferability, or program development for populations thought to be vulnerable to various psychological phenomena. With only two studies designating a non-training control group and none with reported randomization, the magnitude of the effect for relaxation and self-regulation provided by SART itself remains unclear. The lack of reported randomization may stem from the studies relying on convenience samples from clinical and university settings, and nonrandomized targeted activities with support groups for mothers, special needs children, mothers with special needs children, and children attending schools or daycare activities. The exception is the large sample size and quasi-randomized sampling inherent to voluntary participation in the SART activity at temporary housing meeting rooms in the disaster support application by Ki (2015b). Even though this design and effort includes participants of all ages and addresses aspects of population representativeness, it also falls under the category of studies lacking the randomized assignment of participants with a control group required to discriminate training-related effects. Thus, the most pressing limitation is the overall lack of randomized controlled designs in research studies of SART. Ecological validity is also an area of concern, as the overwhelming majority of participants have been Japanese, which limits the generalizability of these findings.

In addition, while there have been a few follow-up reports in previous studies, the duration of the therapeutic effect, assessment of home practice, and precision of the effect of the training apart from a support context remain unclear. Heavy reliance on qualitative research methods and clinical case series also leaves many questions about the evidence base unanswered. While these designs allow for thick narrative description and assessment of client reports of change, the lack of countable variables mapping to tractable constructs makes generalizability challenging. In addition, the fact that these research projects have had low representation in peer-reviewed journals represents a major limitation as it could be a source of publication bias.

For the studies that did measure outcomes, the majority of assessments have been self-report questionnaires and psychometric instruments. Only one article provided reliability estimates for the instruments operationalized in the form of Cronbach's alpha, but other modern estimates such as MacDonald's omega have increasingly been endorsed (Dunn et al., 2014). Due to the lack of this reporting, it is difficult to critically assess the quality of the instruments. Some of the psychometric instruments investigated have been widely validated (e.g., POMS, STAI) as they have been meaningfully reported in prior literature published in English and Japanese, but other especially indigenous scales appear to be infrequently utilized and questionably validated. While many of them appear to be ecologically valid for use in Japan, studies on SART should use instruments with a known track record of reliability beyond POMS and STAI and investigate possible confounders so that they may be compared with relaxation techniques worldwide.

### Future directions

A randomized controlled design using SART is a priority for future research. An especially promising avenue might also be one that compares the therapeutic effect or self-regulatory properties of SART with the other well-known body movement methods. The body movement-based paradigm of emotion regulation proposed by Shafir et al. (2015) might be usefully applied for understanding the affective components of the body movements and their modulation. Another study could investigate combinations of other relaxation modalities in tandem with SART, as it is possible that guided imagery, focusing, breathing, or muscle group contraction techniques could be usefully applied to enhance the sense of relaxation or therapeutic effect provided by SART, or vice-versa.

While two studies have used EMG to compare changes in muscle activity from SART relaxation tasks, the precise relationship between subjective sense of relaxation, mood states, and their connection to the conferred motor resonance is unknown, and other objective measurements have yet to be conducted (Mukasa and Ohno, 2016; Ki et al., 2017). Studies incorporating physiological measures such as skin conductance or heart rate variability are recommended. The presence of movement artifacts in the explicitly movement-based approach of SART may make objective measurement difficult, but relaxation states measured via electroencephalography or fMRI may also be a fruitful endeavor for providing fine-grained evidence and overcoming reliance on self-report.

The use of the body as the target of the psychotherapeutic process allows the therapist and client to co-discover and directly work to relax tension, which centralizes the goals of the approach as an advantage. It is possible that SART facilitates aspects of affect labeling vis-à-vis its emphasis on body state labeling in the pre- and post-assessment. Further research is necessary to understand if the way that SART emphasizes body awareness incurs or enhances body state labeling through assessment, and one approach might be to determine its utility in known groups such as those with alexithymia or alexisomia (Kanbara and Fukunaga, 2016).

### Theoretical considerations for SART and the self-regulation of interoceptive signals

A greater diversity in research questions with SART could indicate more about its effect size, assessment, and the phenomenology of body awareness, health competence, or individual differences that influence responses to relaxation that have yet to be evaluated. The difficulties of implementation science and community psychology approaches to determine the appropriate evidence base underscores the need to clarify the precise mechanism of change that SART provides going forward.

Incorporating ongoing research on theories of body awareness such as interoceptive sensibility or dimensions of self-related embodiment could provide the framework SART needs to measure degrees of change, if any exist, from the use and mastery of the tool as a self-regulatory skill. Interoceptive awareness is posited to be crucial for the self-control of behavior for various circumstances that involve health and disease (Bornemann et al., 2014). As SART was born out of Dohsa-hou movement tasks, it seems plausible that SART shares a mechanism of body awareness facilitation that has been established in prior literature on Dohsa-hou (Fujino, 2012, 2013). While still unknown, the process may be akin to an instantaneous sense of interoceptive awareness, and studies could explore the ability for SART to augment or otherwise affect positive body awareness through measures such as the Multidimensional Assessment of Interoceptive Awareness (MAIA; Mehling et al., 2012; Mehling, 2016) or aspects of the Embodied Sense of Self Scale (ESSS; Asai et al., 2016). While it is theorized to be less volitionally attending, it remains to be seen if SART can enhance attention to interoceptive sensations via the MAIA or the ESSS and improve self-regulation in the same way as other bodymind practices, such as body scan training (Bornemann et al., 2014; Farb et al., 2015). It seems plausible that the self-regulation afforded by SART is one that allows the user to relax their body on their own to help overcome the homeostatic dysregulation caused by disruptive events (Kanbara and Fukunaga, 2016).

## Conclusion

SART is a tool and program of movement tasks that utilizes stress management and process-scaffolding psychoeducation to empower users to regulate tension in the body, improve mood, and attenuate sensations to the here-and-now. From its use as a clinical and stress management tool for state-based mood regulation, to its application as a community program for adopting a bi-annual check-up habit for mental health in tsunami-affected areas of Iwate Prefecture in Japan, SART shows promise as a bodymind approach that can empower users to physically experience changes in psychological states, such as mood, stress, and body awareness, and act on their own to modulate them. Critical assessment has revealed that randomized controlled designs, physiological measurements, assessments of home practice, incorporation of reliable operationalized instruments, and stronger attachment to theories of body awareness and relaxation through constructs such as interoceptive awareness are necessary for future studies to overcome challenges in research on the method. The bodymind approach of SART may have emerged in Japan, but by dint of its universal therapeutic target of the human body and goal of achieving a state of relaxation against the stress response, it seems primed for meaningful translation to the toolkits for self-regulation utilized elsewhere. Professional development in SART for health care specialists exists in Japan and consists of workshops and case study presentations, but is still being refined. Due to the relative novelty of the approach, a notable limitation that must be acknowledged in this review is its major reliance on non-peer-reviewed university repository articles that are unavailable in English and tied to only a handful of institutions, and thus may be subject to publication bias. In addition, this review was written in coordination with the creator and major practitioners of the approach to ensure an accurately translated initial portrayal and appropriate channel of attribution to their original work, and thus does not represent a wholly independent evaluation. Therefore, the authors hope that by bringing SART from Japan to the world readership at-large, this bodymind approach to resilience can be explored, improved, and corroborated independently through broader and more rigorous study designs and applications by future researchers.

## Author contributions

HO, HK, and YH: Access to the articles and original documents by the creators of the approach, permission to use figures from published works, and revisions of this manuscript were provided by the co-authors; RK: These were reviewed, translated, and summarized by the corresponding author.

### Conflict of interest statement

The authors declare that the research was conducted in the absence of any commercial or financial relationships that could be construed as a potential conflict of interest.

## References

[B1] Abidin R. R. (1983). Parental Stress Index. Charlottesville, VA: Pediatric Psychology Press.

[B2] Aramaki M. Muto T. (2008). Factors related to negative and positive feelings about child-rearing: a survey of mothers of young children. Japan. J. Dev. Psychol. 19, 87–97. 10.11201/jjdp.19.87

[B3] Asai T. Kanayama N. Imaizumi S. Koyama S. Kaganoi S. (2016). Development of embodied sense of self scale (ESSS): exploring everyday experiences induced by anomalous self-representation. Front. Psychol. 7:1005. 10.3389/fpsyg.2016.01005 27458403 PMC4932106

[B4] Bornemann B. Herbert B. M. Mehling W. E. Singer T. (2014). Differential changes in self-reported aspects of interoceptive awareness through 3 months of contemplative training. Front. Psychol. 5:1504. 10.3389/fpsyg.2014.01504 25610410 PMC4284997

[B5] Buchanan P. A. Ulrich B. D. (2001). The Feldenkrais Method®: a dynamic approach to changing motor behavior. Res. Q. Exerc. Sport 72, 315–323. 10.1080/02701367.2001.10608968 11770781

[B6] Burklund L. J. Creswell J. D. Irwin M. Lieberman M. (2014). The common and distinct neural bases of affect labeling and reappraisal in healthy adults. Front. Psychol. 5:221. 10.3389/fpsyg.2014.00221 24715880 PMC3970015

[B7] Calì G. Ambrosini E. Picconi L. Mehling W. Committeri G. (2015). Investigating the relationship between interoceptive accuracy, interoceptive awareness, and emotional susceptibility. Front. Psychol. 6:1202. 10.3389/fpsyg.2015.01202 26379571 PMC4547010

[B8] Caldwell C. Victoria H. K. (2011). Breathwork in body psychotherapy: towards a more unified theory and practice. Body Mov. Dance Psychother. 6, 89–101. 10.1080/17432979.2011.574505

[B9] Chervenkova V. (2015). Application of Dohsa-hou in Japan and Abroad 5.2. Introduction and implementation of Dohsa-hou in Bulgaria–the main challenges, outcomes and perspectives, in Introduction to Dohsa-hou–An Integrated Japanese Body-Mind Therapy, 86.

[B10] Chervenkova V. (2017). Japanese Psychotherapies: Silence and Body-Mind Interconnectedness in Morita, Naikan and Dohsa-hou. Singapore: Springer.

[B11] Craig A. D. (2002). Opinion: how do you feel? Interoception: the sense of the physiological condition of the body. Nat. Rev. Neurosci. 3, 655 10.1038/nrn894 12154366

[B12] Dadkhah A. (1998). Body consciousness in Dohsa-hou, a Japanese psychorehabilitative program. Percept. Mot. Skills 86, 411–417. 10.2466/pms.1998.86.2.411 9638742

[B13] Damasio A. R. (1994). Descartes' Error: Emotion, Rationality and the Human Brain. New York, NY: Putnam.

[B14] de Jong M. Lazar S. W. Hug K. Mehling W. E. Hölzel B. K. Sack A. T.. (2016). Effects of mindfulness-based cognitive therapy on body awareness in patients with chronic pain and comorbid depression. Front. Psychol. 7:967. 10.3389/fpsyg.2016.00967 27445929 PMC4927571

[B15] Dunn T. J. Baguley T. Brunsden V. (2014). From alpha to omega: a practical solution to the pervasive problem of internal consistency estimation. Br. J. Psychol. 105, 399–412. 10.1111/bjop.12046 24844115

[B16] Dusek J. A. Benson H. (2009). Mind-body medicine: a model of the comparative clinical impact of the acute stress and relaxation responses. Minn. Med. 92, 47. 19552264 PMC2724877

[B17] Farb N. Daubenmier J. Price C. J. Gard T. Kerr C. Dunn B. D.. (2015). Interoception, contemplative practice, and health. Front. Psychol. 6:763. 10.3389/fpsyg.2015.00763 26106345 PMC4460802

[B18] Fujino H. (2012). Effects of Dohsa-hou relaxation on body awareness and psychological distress. Jpn. Psychol. Res. 54, 388–399. 10.1111/j.1468-5884.2012.00517.x

[B19] Fujino H. (2013). Subjective experience of Dohsa-hou relaxation: a qualitative study. Asia Pac. J. Couns. Psychother. 4, 66–75. 10.1080/21507686.2013.775170

[B20] Gard T. Noggle J. J. Park C. L. Vago D. R. Wilson A. (2014). Potential self-regulatory mechanisms of yoga for psychological health. Front. Hum. Neurosci. 8:770. 10.3389/fnhum.2014.00770 25368562 PMC4179745

[B21] Garland E. L. Geschwind N. Peeters F. Wichers M. (2015). Mindfulness training promotes upward spirals of positive affect and cognition: multilevel and autoregressive latent trajectory modeling analyses. Front. Psychol. 6:15. 10.3389/fpsyg.2015.00015 25698988 PMC4313604

[B22] Gendlin E. T. (1991). On emotion in therapy, in Emotion, Psychotherapy and Change, eds Safran J. D. Greenberg L. S. (New York, NY; London: Guilford), 255–279.

[B23] Gonçalves L. C. de Souza Vale R. G. Barata N. J. F. Varejão R. V. Dantas E. H. M. (2011). Flexibility, functional autonomy and quality of life (QoL) in elderly yoga practitioners. Arch. Gerontol. Geriatr. 53, 158–162. 10.1016/j.archger.2010.10.028 21167613

[B24] Haggard P. (2017). Sense of agency in the human brain. Nat. Rev. Neurosci. 18, 196–207. 10.1038/nrn.2017.14 28251993

[B25] Harada S. (2016). When immovable bodies start to move–Dohsa-hou for children with cerebral palsy, in Introduction to Dohsa-hou–An Integrated Japanese Body-Mind Therapy, 19.

[B26] Harada S. Teruta E. (2016). Application of Dohsa-hou in Japan and abroad 5.1, in Dohsa-hou camps and monthly meetings in Japan. Introduction to Dohsa-hou–An Integrated Japanese Body-Mind Therapy, 78.

[B27] Imura O. (2016). The birth and development of Dohsa-hou, in Introduction to Dohsa-hou–An Integrated Japanese Body-Mind Therapy, 19.

[B28] Imura O. Furukawa T. Fujino H. Sugao S. Chervenkova V. Teruta E. (2016). Introduction to Dohsa-hou: an Integrated Japanese Body-Mind Therapy. Available online at: www.hus.osaka-u.ac.jp/en/book/911

[B29] Ishimaru H. Ki H. (2012). Clinical psychological study for parenting support focused on mother's empowerment: improvement of self-trust by SART. Clin. Psychol. Bull. 9, 1–9.

[B30] Kaminoff L. Matthews A. Ellis S. (2007). Yoga Anatomy. Champaign, IL: Human Kinetics.

[B31] Kanbara K. Fukunaga M. (2016). Links among emotional awareness, somatic awareness and autonomic homeostatic processing. Biopsychosoc. Med. 10, 16. 10.1186/s13030-016-0059-3 27175214 PMC4863353

[B32] Ki H. (2008). A study of the relaxation in Dohsa-hou: therapeutic support focused in self-activeness. Clin. Psychol. Bull. 5, 75–83. [In Japanese].

[B33] Ki H. (2015a). The Training and Specialization of the Clinical Psychologist: Lessons from Disaster Support. Fukuoka: Clinical Psychology Center of Fukuoka Jo Gakuin University. [In Japanese].

[B34] Ki H. (2015b). Applications of relaxation to stress management II: implementation of self-active relaxation therapy. Hiroshima Psychol. Res. 14, 12–16. [In Japanese]. 10.15027/39584

[B35] Ki H. Ohno H. (2006). The experiencing process in self-active relaxation therapy. Clin. Psychol. Bull. 3, 1–9. [In Japanese].

[B36] Ki H. Ohno H. (2008). The case study of Self-Active Relaxation Therapy (SART) for people with physical, developmental and severely mental and physical disabilities. Int. J. Psychol. 43, 408. [In Japanese].

[B37] Ki H. Doi T. Ohno H. (2013). Stress management skill in special needs education. Clin. Psychol. Bull. 10, 1–13. [In Japanese].

[B38] Ki H. Inoue Y. Ohno H. (2016). Psychological effect of stress management by SART from the viewpoint of self-esteem and self-control. Clin. Psychol. Bull. 13, 19–24. [In Japanese].

[B39] Ki H. Kishikawa N. Ohno H. (2017). A comparative study of single-SART and cooperative-SART. Clin. Psychol. Bull. 14, 55–74. [In Japanese].

[B40] Ki H. Ohno H. Arikawa M. Mukasa R. (2014). Clinical psychological approach to the disaster recovery support of the Great East Japan Earthquake. Clin. Psychol. Bull. 11, 53–60. [In Japanese].

[B41] Kolt G. S. McConville J. C. (2000). The effects of a Feldenkrais® awareness through movement program on state anxiety. J. Bodyw. Mov. Ther. 4, 216–220. 10.1054/jbmt.2000.0179

[B42] Konno Y. (1999). The lateral effects of Dohsa-method relaxation on visual and auditory responses. Jpn. Psychol. Res. 41, 193–202. 10.1111/1468-5884.00119

[B43] Konno Y. (2016). Psychotherapeutic approach of Dohsa-hou in Japan. J. Spec. Educ. 5, 11–17. 10.6033/specialeducation.5.11

[B44] Kubota N. (2000). An analysis of the therapeutic process of Dosa therapy, Doctoral dissertation, Michigan State University, School of Social Work. Retrieved from ProQuest Dissertations and Theses, 203 (304626933). Order No. 9985413.

[B45] Linden W. (1994). Autogenic training: a narrative and quantitative review of clinical outcome. Appl. Psychophysiol. Biofeedback 19, 227–264. 10.1007/BF01721069 7811786

[B46] Little P. Lewith G. Webley F. Evans M. Beattie A. Middleton K. (2008). Randomised controlled trial of Alexander technique lessons, exercise, and massage (ATEAM) for chronic and recurrent back pain. BMJ 337:a884 10.1136/bmj.a884 18713809 PMC3272681

[B47] Lorr M. McNair D. Heuchert J. W. P. Droppleman L. F. (1982). Profile of Mood States. San Diego, CA: Educational and Industrial Testing Service.

[B48] Matsufuji M. Ki H. (2011). Case study of psychological rehabilitation of at-risk children and the formation of good mother-child relations using SART. Clin. Psychol. Bull. 8, 35–44. [In Japanese].

[B49] Mehling W. (2016). Differentiating attention styles and regulatory aspects of self-reported interoceptive sensibility. Philos. Trans. R. Soc. B 371:20160013. 10.1098/rstb.2016.0013 28080970 PMC5062101

[B50] Mehling W. E. Hamel K. A. Acree M. Byl N. Hecht F. M. (2005). Randomized, controlled trial of breath therapy for patients with chronic low-back pain. Altern. Ther. Health Med. 11:44. 16053121

[B51] Mehling W. E. Price C. Daubenmier J. J. Acree M. Bartmess E. Stewart A. (2012). The multidimensional assessment of interoceptive awareness (MAIA). PLoS ONE 7:e48230. 10.1371/journal.pone.0048230 23133619 PMC3486814

[B52] Mori M. Ohno H. (2017). The study of psychological support for handicapped persons by using self-active relaxation therapy: self-awareness of people with developmental disabilities. Clin. Psychol. Bull. 14, 35–45. [In Japanese].

[B53] Motomura H. Ohno H. (2017). Effect of stress management by SART for mothers of children with tendency of developmental disorders. Clin. Psychol. Bull. 14, 75–84. [In Japanese].

[B54] Mukasa R. Ohno H. (2016). Psychophysiological study of self-active relaxation therapy by use of electromyography. Clin. Psychol. Bull. 13, 43–54. [In Japanese].

[B55] Murakami R. Ki H. (2011). Research on psychological support for children in foster homes: an effect of SART. Fukuoka Jo Gakuin University: Clinical Psychology Bulletin 8, 45–52. [In Japanese].

[B56] Naruse G. (1973). Shinri Rehabilitation [Psychological Rehabilitation]. Tokyo: Seishin-Shobo.

[B57] Naruse G. (1988). Jiko-Control-hou [Self-control method]. Tokyo: Seishin-Shobo.

[B58] Naruse G. (1997). The clinical Dohsa-hou as psychotherapy. Jpn. J. Rehabil. Psychol. 25, 9–16.

[B59] Niina R. Sakata S. Yatomi N. Honma A. (1990). Development of the psychological stress response scale. [Shinshin-Igaku]. Japan. J. Psychosom. Med. 30, 29–38. [In Japanese]. 10.15064/jjpm.30.1_29

[B60] Nozawa M. (1989). Oyagyou sutoresusu ni kansuru kisoteki [Study on parenting stress]. Annu. Bull. 15, 35–56. [In Japanese].

[B61] Ohno H. (2005). SART: Self-Active Relaxation Therapy. Fukuoka: Kyushu University Press.

[B62] Ohno H. (2006). Theoretical development of self-active relaxation therapy. Jpn. J. Rehabil. Psychol. 33, 41–54. [In Japanese].

[B63] Ohno H. (2007). Basic SART Techniques with Q&A. Fukuoka: NPO Psychological Rehabilitation Center. [In Japanese].

[B64] Ohno H. (2010). What is ‘self-active’ in self-active relaxation therapy? Clin. Psychol. Bull. 7, 1–19. [In Japanese].

[B65] Ohno H. (2015). Applications of relaxation to stress management I: principles of self-active relaxation therapy. Hiroshima Psychol. Res. Bull. 14, 3–11. [In Japanese with English abstract]. 10.15027/39583

[B66] Ohno H. Dadkhah A. Noi M. Ohzuru K. (2004). Self-Active Relaxation Therapy (SART) in Dohsa-hou. Jpn. J. Rehabil. Psychol. 32, 1–13.

[B67] Okuzono K. Ki H. (2010). A study of child care support with self-active relaxation therapy. Clin. Psychol. Bull. 7, 11–19. [In Japanese].

[B68] Paulus M. P. Stein M. B. (2010). Interoception in anxiety and depression. Brain Struct. Funct. 214, 451–463. 10.1007/s00429-010-0258-9 20490545 PMC2886901

[B69] Payne P. Crane-Godreau M. A. (2015). The preparatory set: a novel approach to understanding stress, trauma, and the bodymind therapies. Front. Hum. Neurosci. 9:178. 10.3389/fnhum.2015.00178 25883565 PMC4381623

[B70] Payne P. Fiering S. Leiter J. C. Zava D. T. Crane-Godreau M. A. (2017). Effectiveness of a novel qigong meditative movement practice for impaired health in flight attendants exposed to second-hand cigarette smoke. Front. Hum. Neurosci. 11:67. 10.3389/fnhum.2017.00067 28270757 PMC5318411

[B71] Sakurai S. Takano S. (1985). A new self-report of intrinsic versus extrinsic motivation toward learning in children. Tsukuba Psychol. Res. 7, 43–54. [In Japanese].

[B72] Schultz J. H. (1932). Das Autogene Training: Konzentrative Selbstentspannung. Leipzig: Thieme.

[B73] Schulz A. Vögele C. (2015). Interoception and stress. Front. Psychol. 6:993. 10.3389/fpsyg.2015.00993 26257668 PMC4507149

[B74] Shafir T. Tsachor R. P. Welch K. B. (2015). Emotion regulation through movement: unique sets of movement characteristics are associated with and enhance basic emotions. Front. Psychol. 6:2030. 10.3389/fpsyg.2015.02030 26793147 PMC4707271

[B75] Shimada H. Togasaki Y. Sakano Y. (1994). Development of stress response scale for children. Japan. J. Health Psychol. 7, 46–58. [In Japanese]. 10.11560/jahp.7.2_46

[B76] Stahl J. E. Dossett M. L. LaJoie A. S. Denninger J. W. Mehta D. H. Goldman R.. (2015). Relaxation response and resiliency training and its effect on healthcare resource utilization. PLoS ONE 10:e0140212. 10.1371/journal.pone.0140212 26461184 PMC4603901

[B77] Susaki Y. Anii A. (2013). The examination of validity, reliability, and factor structure of self-esteem scale by Rosenberg for elementary and junior high school students. Life Needs Exp. Learn. Res. 13, 93–98. [In Japanese].

[B78] Takeo E. Ohno H. Ki H. (2016). Study of psychological support for aged persons by use of self-active relaxation therapy. Clin. Psychol. Bull. 13, 1–9. [In Japanese].

[B79] Terasaki F. (2009). An attempt to make the scale of measure ‘self-trust’. Kyushu Univ. Psychol. Res. 10, 159–166.

[B80] Tsakiris M. Critchley H. (2016). Interoception beyond homeostasis: affect, cognition and mental health. Philos. Trans. R. Soc. B 371:20160002. 10.1098/rstb.2016.0002 28080961 PMC5062092

[B81] Utsunomiya Y. Ohno H. (2012). Research on stress management for elementary school students: utilizing single-SART. Clin. Psychol. Bull. 9, 11–17. [In Japanese].

[B82] Van der Kolk B. (2014). The Body Keeps the Score: Mind, Brain and Body in the Transformation of Trauma. New York, NY: Viking.

[B83] Vancampfort D. Correll C. U. Scheewe T. W. Probst M. De Herdt A. Knapen J.. (2013). Progressive muscle relaxation in persons with schizophrenia: a systematic review of randomized controlled trials. Clin. Rehabil. 27, 291–298. 10.1177/0269215512455531 22843353

[B84] Woodman J. P. Moore N. R. (2012). Evidence for the effectiveness of Alexander Technique lessons in medical and health-related conditions: a systematic review. Int. J. Clin. Pract. 66, 98–112. 10.1111/j.1742-1241.2011.02817.x 22171910

[B85] Yamamoto K. Hukui M. (2009). Attempt to make a scale of junior high school students' response to stresses. [Kango hoken kagaku kenkyu-shi]. J. Nurs. Health Sci. Res. 9, 71–77. [In Japanese].

[B86] Yasukouchi R. Ki H. (2012). Case study of SART for improvement of self-esteem in children with developmental disabilities. Clin. Psychol. Bull. 9, 45–51. [In Japanese].

[B87] Yokoyama K. Araki S. Kawakami N. Takeshita T. (1990). Production of the Japanese edition of profile of mood states (POMS): assessment of reliability and validity. [Nihon koshu eisei zasshi]. Japan. J. Public Health 37, 913–918. [In Japanese]. 2132363

