# Supplementary material for: Self-Active Relaxation Therapy (SART) and Self-Regulation: A Comprehensive Review and Comparison of the Japanese Body Movement Approach
Source: Front Hum Neurosci. 2018 Feb 8;12:21. doi: 10.3389/fnhum.2018.00021 (PMC5809433; doi:10.3389/fnhum.2018.00021)
Supplement: Supplementary file 1 [file DataSheet1.pdf]

Supplementary Figure 1. Pre- and post-assessment range-of-motion check. SART begins with an intake procedure and range-of-motion assessment. While seated, the client laterally veers his or her outstretched arms to the left and right on both of sides of the body. This process is repeated as a post-assessment and the client compares their former stretch limitation with their latter one. The client should notice a change in their range of motion as a result of their relaxed muscles from the SART suite of movement tasks. Adapted from *SART: Self-Active Relaxation Therapy* by Hiroyuki Ohno (2005), Fukuoka: Kyushu University Press. Copyright 2005 by Hiroyuki Ohno. Adapted with permission.

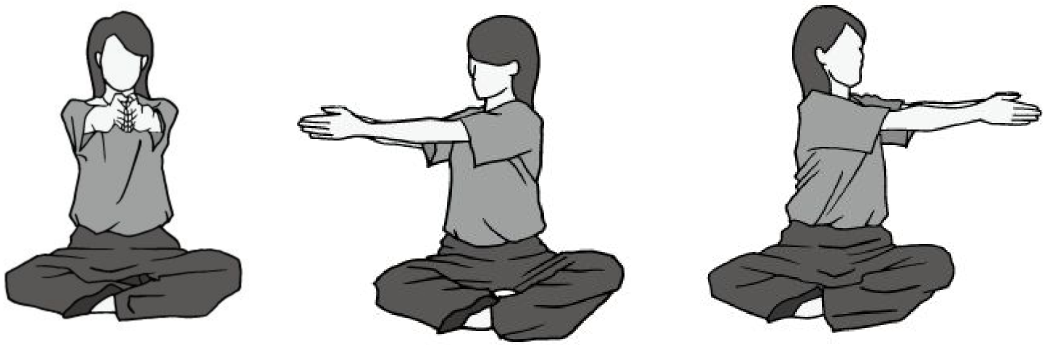

Supplementary Figure 2. The basic posture of SART. The client conforms to a lateral recumbent position, usually with the knee from the overlapping leg bent and brought forward toward the waist for the support of a neutral spine. Adapted from *SART: Self-Active Relaxation Therapy* by Hiroyuki Ohno (2005), Fukuoka: Kyushu University Press. Copyright 2005 by Hiroyuki Ohno. Adapted with permission.

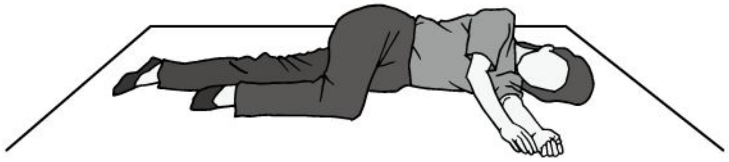

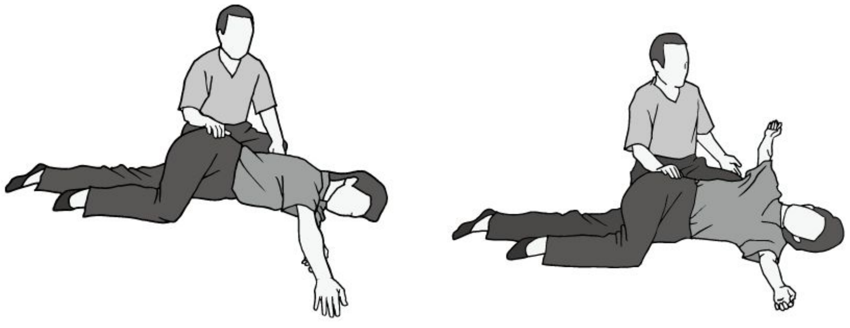

(1) *Horizontal abduction of the shoulder joint.*

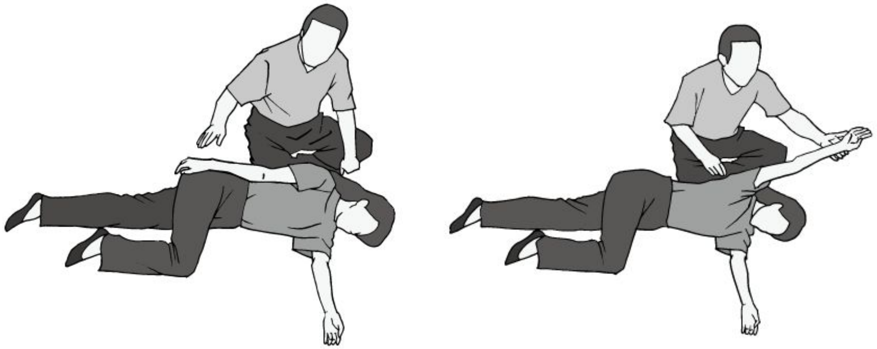

(2) *Abduction of the shoulder joint.*

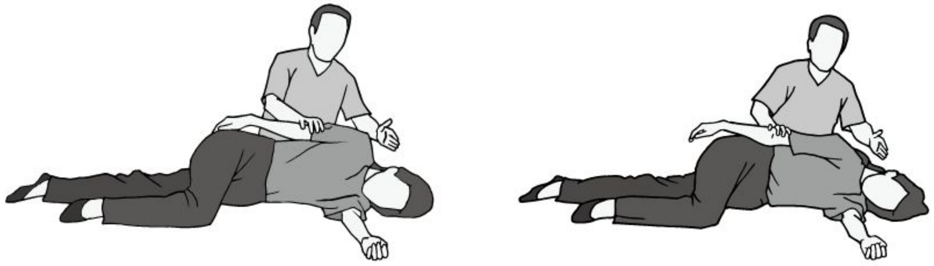

(3) *Elevation and depression of the shoulder joint.*

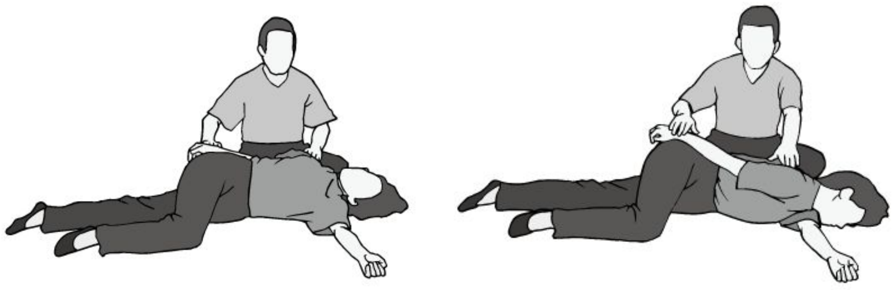

(4) *Trunk rotation with flexion and extension of the shoulder joint.*

APPENDIX 1. The upper body movements of System 1 in SART. (1) depicts an anteroposterior reach, with (2) encompassing an overhead arm raise along the longitudinal axis. The movement in (3) is designed to raise and lower the shoulder, and (4) to open and close the chest. The movements are not coordinated with explicit breathing instructions except to open the diaphragm and generate a wider range of motion in (1) and (4), and guided attention is not emphasized with the exception of advised discrimination of neck and shoulder muscle groups in (3). Adapted from *SART: Self-Active Relaxation Therapy* by Hiroyuki Ohno (2005), Fukuoka: Kyushu University Press. Copyright 2005 by Hiroyuki Ohno. Adapted with permission.

APPENDIX 2. The lower body movements of System 2 in SART. The movements in (5) are accompanied by medial and lateral rotation of the toes. The therapist encourages the client to continue until they feel the stretch in (6), guides the movement in (7), and contributes to the flexion in (8). Adapted from *SART: Self-Active Relaxation Therapy* by Hiroyuki Ohno (2005), Fukuoka: Kyushu University Press. Copyright 2005 by Hiroyuki Ohno. Adapted with permission.

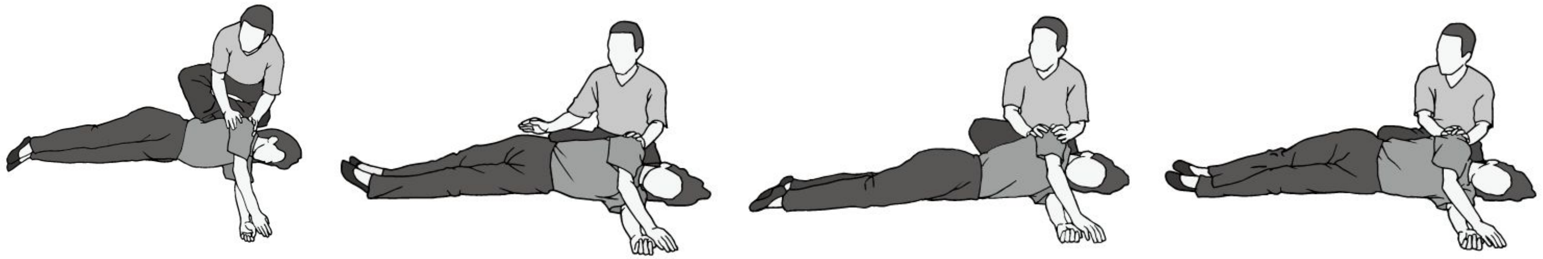

(5) *Trunk rotation at the hip joint.*

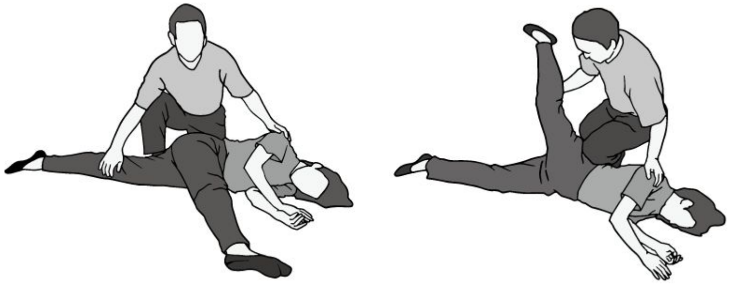

(6) *Horizontal abduction of the hip joint.*

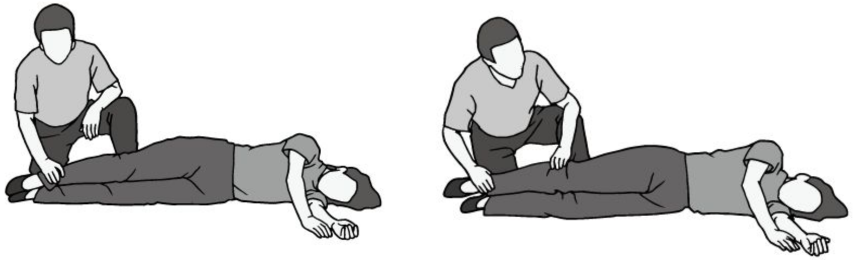

(7) *Elevation and depression of the hip joint.*

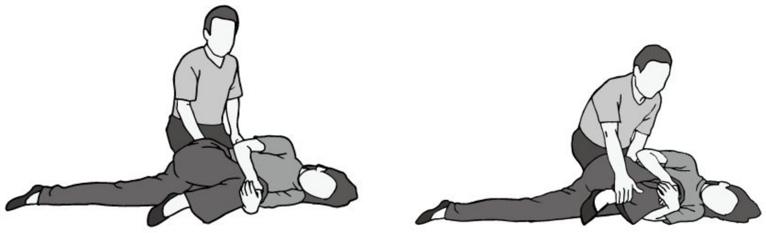

(8) *Elevation of the knee and flexion of the hip joint.*

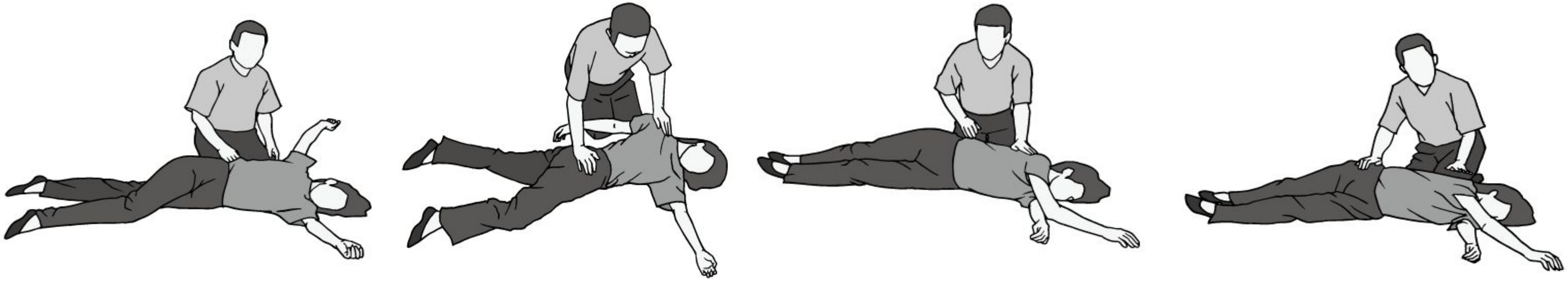

(9) *Trunk rotation at the hip joint with horizontal abduction at the shoulder joint.*

APPENDIX 3. The multi-sectional movements of System 3 in SART which focus on stretches across the whole body. The movements in (9) integrate the horizontal abduction of the shoulder in System 1 and trunk rotation of System 2, with the therapist supporting the form and intervening to help the client achieve a deeper stretch. Adapted from *SART: Self-Active Relaxation Therapy* by Hiroyuki Ohno (2005), Fukuoka: Kyushu University Press. Copyright 2005 by Hiroyuki Ohno. Adapted with permission.
